# Associations Between Adolescent Problematic Internet Use and Relationship Problems in Chinese Families: Findings from a Large-scale Survey

**DOI:** 10.2196/35240

**Published:** 2022-10-24

**Authors:** Alimila Hayixibayi, Esben Strodl, Wei-Qing Chen, Adrian B Kelly

**Affiliations:** 1 School of Psychology and Counselling, Faculty of Health Queensland University of Technology Brisbane Australia; 2 Department of Epidemiology, School of Public Health Sun Yat-sen University Guangzhou China

**Keywords:** problematic internet use, parental bonding, verbal conflict, emotional abuse, physical abuse, adolescent, teenager, internet use, internet usage, abuse, abusive, conflict, family, parental bond, student, Asia, China, parent-child bond, high school, child

## Abstract

**Background:**

Problematic internet use (PIU) is prevalent among Chinese adolescents. There is a need to better understand how the quality of parent-adolescent relationship is associated with adolescent PIU to guide the development of effective prevention and early intervention programs.

**Objective:**

This study aims to evaluate parent-adolescent conflict and parenting styles as potential risk factors associated with adolescent PIU.

**Methods:**

A sample of 6552 students (aged 10-19 years) from 22 schools in Guangdong, China, was recruited. The participants completed self-report questionnaires measuring their perceptions of conflict with their parents (involving verbal conflict, emotional abuse, and physical abuse) as well as their perceptions of their parents’ parenting styles (including parental care and parental control as measured by the Parental Bonding Inventory), and PIU using the Adolescent Pathological Internet Use Scale. Grade level and gender were examined as moderators of these associations.

**Results:**

Using multiple regression analyses, we found that greater mother-adolescent conflict, father-adolescent conflict, and parental control, and lower levels of parental care, were associated with higher levels of adolescent PIU (*P*<.001). The association between mother-adolescent conflict and PIU was stronger in older students than in younger students (*P*=.04), whereas the association between father-adolescent conflict and PIU was stronger in male students than in female students (*P*=.02). Compared with those who reported no mother-adolescent conflict, participants who experienced verbal conflict and emotional abuse, but not physical abuse from their mothers, reported higher levels of PIU (*P*<.001). Compared with those who reported no father-adolescent conflict, participants who experienced verbal conflict, emotional abuse, and physical abuse from their fathers had significantly higher levels of PIU (*P*<.001, *P*<.001, and *P*=.02, respectively).

**Conclusions:**

These findings point to the value of interventions to reduce parental verbal conflict, emotional abuse, and physical abuse, and to increase positive parenting styles, to lower the risk of PIU in Chinese adolescents.

## Introduction

Problematic internet use (PIU) involves a strong compulsion to use the internet to the extent that it creates significant problems for the user, including social isolation, mental health concerns, and academic performance problems [1-3]. While internet use is beneficial in many aspects, for a proportion of adolescents, internet use dominates daily life and is associated with negative psychological, social, and physical impacts, resulting in PIU. Given that adolescence has been shown to be a vulnerable period for a range of mental health problems [4], it is important to identify the risk/protective factors of PIU for early prevention. PIU is reported by around 9% of Chinese adolescents [5]. A systematic review found that the main risk and protective factors for adolescent PIU that have been investigated appear to be individual factors (eg, psychopathology, academic disposition, or personal attributes), with the authors calling for more research exploring contextual risk/protective factors (eg, family, peer, and school relationships) and internet activity–related factors (ie, internet application used) [6]. Given the emerging evidence that family relationships are likely to be an important contextual risk/protective factor for adolescent PIU [7], this study aims to contribute to the literature by further investigating potential family relationships that may be risk and protective factors of adolescent PIU in China.

Based on Attachment Theory [8,9], adolescent’s emotional security is largely influenced by the quality of the parent-child relationship, with less care from parents and feeling more controlled by parents linked to higher emotional insecurity, which in turn can increase the risk of adolescent problematic behaviors [10]. This is because adolescents may develop internal working models of themselves as unworthy of love, and of others as unreliable in providing emotional security, based on their interactions with attachment figures (eg, parents) [11]. From this perspective, adolescent PIU can be seen as a maladaptive coping strategy to manage an adolescent’s distress, or unmet emotional needs, arising from parent-child relationships [12]. For example, Yu et al [13] investigated the prospective relations between parental control and maladjustment in Chinese adolescents. Their results found that high levels of paternal control were predictive of depressed mood, anxiety, and aggression among Chinese adolescents. Similarly, Siomos et al [14] found that parental control is a parenting style that is prospectively and positively associated with PIU after taking into account parental online safety practices. In addition, adolescents with PIU report experiencing a greater lack of emotional warmth and feelings of rejection from parents than adolescents without PIU [15]. Moreover, Faltýnková et al [16] found that a parenting style that exhibited more warmth was a protective factor, while parental control was a risk factor, for adolescent PIU after considering other family factors such as parental monitoring. Such research highlights the importance of examining the relationships between the 2 main dimensions of parenting style that have been shown to be associated with adolescents bonding to their parents (parental control and parental care) and the presence of adolescent PIU. However, previous studies have not examined the unique contribution of multiple aspects of parenting styles to Chinese adolescents’ PIU. Therefore, more research is needed to investigate the independent effects of parenting styles, such as parental warmth/care and control, on PIU among Chinese adolescents.

In addition to parental bonding, there is evidence that other specific parent-adolescent interactions may be important risk factors for adolescent maladaptive coping and mental health problems. For instance, longitudinal studies have demonstrated that family conflict is consistently associated with poor adolescent mental health [17,18]. More specifically, emerging evidence shows that parent-adolescent conflict is likely to predict PIU. For example, Lo et al [19] reported that more harsh parenting (defined by physical abuse and verbal aggression) was related to higher PIU in a sample of 1204 Chinese adolescents from 7th to 9th grade [19]. However, there is little research exploring whether conflict with parents is an additional risk factor for PIU after controlling for parental bonding. Only a recent study has found that both higher levels of parental control and physical/verbal abuse by parents (forms of parent-adolescent conflict) were associated with higher levels of internet gaming disorder (a related construct to PIU) among 2666 Chinese students aged 11-13 years [20]. As such, there is emerging empirical evidence indicating that there may be multiple parent-adolescent interactions that are independent risk and protective factors for adolescent PIU. Gaining a more comprehensive understanding of the range and relative strength of these independent risk and protective factors will help guide the development of more targeted family-based intervention programs for adolescent PIU.

Furthermore, given that the prevalence of PIU varies by gender and grade [21], it is possible that association between risk and protective factors and PIU may be moderated by gender and age. In terms of gender as a potential moderator, there is evidence that adolescent girls tend to be more reactive to interpersonal conflict, leading to a greater use of maladaptive coping in response to interpersonal conflict, compared with adolescent boys [9]. Similarly, previous research has found that the positive associations of mother-adolescent conflict and teacher-adolescent conflict with adolescent depressive symptoms were stronger in female students than in male students [22]. We, therefore, anticipate that the associations between family-based relationships (ie, parent-adolescent conflict and parenting styles) and PIU will be stronger in female adolescents than in male students. In terms of grade level as a potential moderator, previous research has reported that older adolescents are likely to have higher PIU [23]. Older adolescents are more inclined to anxiety and depressed mood than younger adolescents, and previous research has found that the association of peer-adolescent conflict with PIU is stronger in older adolescents than in younger adolescents [24]. Therefore, we expect that the effects of family-based relationships on PIU will be stronger in older adolescents than in younger adolescents.

In addition, this study aims to explore the relations between specific forms of parent-adolescent conflict such as verbal conflict, emotional and physical abuse, and adolescent PIU. Parent-adolescent conflict has been considered as a risk factor for adolescent maladjustment [25], including adolescent PIU [26], but it has also been considered as developmentally normal and may be an important social developmental experience for acquiring expressive and problem-solving skills [27]. Moreover, there is some evidence that different forms of adolescent conflict with parents have varying effects on adolescent mental health [28,29]. It is therefore possible that different forms of parent-adolescent conflict may produce different levels of risk for adolescent PIU. Associated with this, there is evidence that father-adolescent relationships may have different associations with adolescent PIU than mother-adolescent relationships. For example, Liu et al [30] found that lower levels of father-adolescent relationships (defined by dimensions such as perceived emotional closeness and communication), but not mother-adolescent relationships, were associated with higher levels of PIU [30]. Hsieh et al [31] also discovered that paternal but not maternal physical abuse predicted PIU among Chinese fourth-grade students. As such, we will compare the associations between different forms of adolescent-parent conflict and PIU separately for mothers and fathers.

In summary, the key hypotheses and exploration aims for this study are as follows:

- Mother-adolescent conflict and father-adolescent conflict will be positively associated with PIU after controlling for gender, grade level, maternal and paternal education level, family structure, and academic rank.
- After accounting for the aforesaid variables, higher levels of parental care will be associated with lower levels of PIU, whereas higher levels of parental control will be associated with higher levels of PIU.
- These associations will be moderated by gender and grade in that the effects for parenting styles and parent-adolescent conflict will be stronger for females than for males, and stronger for older students than for younger students.
- Compared with adolescents experiencing no mother-/father-adolescent conflict, those experiencing verbal conflict, emotional abuse, or physical abuse from their mothers/fathers will experience varying effects on PIU.

## Methods

### Participants

The sample was recruited from schools in Longhua District, Shenzhen, Guangdong, China. In phase 1, 22 out of 59 schools from 4 administrative districts were randomly selected. In phase 2, 3 classes were selected from each grade out of grades 5, 6, 7, 8, 10, and 11 among the selected schools. Students from grades 9 and 12 were not included as they were in tight study schedules. A total of 6638 students were invited in the study with a response rate of 98.70% (6552/6638). According to the World Health Organization’s recommended age for adolescent [32], we only included participants aged 10-19 years, resulting in 6552 adolescents included in the final analysis.

### Measures

#### Demographics

The demographic variables in our study were age; gender; academic ranking; grade level (primary school including grades 5 and 6, secondary school including grades 7, 8, 10, and 11); family structure (ie, the first category, including those living as nuclear families, and others category, including but not limited to single-parent families or reconstituted families); maternal and paternal education level (9th grade or less, 10–12th grade, and undergraduate degree or above).

#### Problematic Internet Use

Adolescents reported on the degree of PIU using the 5-item Adolescent Pathological Internet Use Scale [2]. This scale includes 38 items, with each item rated on a Likert scale (from 1=“not true at all” to 5=“true all the time”). Higher scores indicate higher PIU. The scale has good convergent validity compared with other commonly used scales (eg, Young’s Internet Addiction Test, Chen Internet Addiction Scale). Its test-retest reliability and internal consistency were also high (0.86 and 0.97, respectively), as reported in a previous study [33]. In our study, the Cronbach α for the scale was .97.

#### Mother-/Father-Adolescent Conflict

This was assessed using the following 3 questions in Mandarin: verbal conflict “In the past 12 months, have you ever had a serious quarrel with your mother/father?”; emotional abuse “In the past 12 months, have you been emotionally punished (eg, being scold, threatened) by your mother/father?”; physical abuse “In the past 12 months, have you ever been physically punished (eg, being forced to stand for some time) by your mother/father?”. Conflict measurement was rated using the 5-point Likert scale (from 0=“never” to 4=“always”), with higher scores indicating higher levels of mother-/father-adolescent conflict. These items have been applied in previous studies on Chinese adolescents [22,34]. The Cronbach α in this study was .64 for mother-adolescent conflict and .74 for father-adolescent conflict.

#### Parenting Styles

Parenting styles are commonly measured using the Parental Bonding Instrument (PBI), which defines optimal parental bonding in terms of a combination of high parental care (eg, warmth, empathy) and low parental control (eg, overprotection, intrusion) [35]. The PBI consists of 20 items and uses a 5-point Likert scale ranging from “strongly agree” to “strongly disagree.” The PBI has 2 subscales (parental care and parental control), for mothers and fathers separately, each with 10 items. Items 1, 4, 5, 6, and 8 score from 0 (strongly agree) to 4 (strongly disagree). Items 2, 3, 7, 9, and 10 score from 4 (strongly agree) to 0 (strongly disagree). Higher scores indicate higher levels of parental care and lower levels of parental control that adolescents perceive. The PBI has 2 subscales (care and control), for mother and father separately, each with 10 items. Examples include “My mother/father appeared to understand my problems and worries” and “My mother/father tried to control everything I did.” A composite α value for each subscale (eg, mother care and father care combined) was reported in previous research [35]. The Cronbach α values for this study (.61 for parental control and .81 for parental care) were comparable with previous research [16] (.65 for parental control and .88 for parental care).

### Procedure

Students completed this survey during class time. All items on the survey were written in Mandarin. Research assistants supervised the survey completion in the absence of the teachers. Participation in the survey was voluntary without any information that can be identified.

### Statistical Analyses

All statistical analyses were performed using SPSS version 25.0 (IBM Corp.). Of the total sample size (N=6552), the percentage of missing data for all variables was less than 6% (386/6552, 5.89%). Missing values were imputed 20 times using multiple imputations in SPSS and the pooled value was used for the results. Pearson correlation was used to examine the bivariate associations between the variables. To examine whether there was a significant difference between schools, a 2-level (individuals nested within schools) linear regression was performed, which indicated that only around 4% of variance was explained at the school level, suggesting that there was low clustering of observations (intraclass correlation <0.05). Hence, multiple linear regression was used to examine the associations between the quality of family relationships and PIU after controlling for the covariates (see hypotheses). The independent variables were transformed into centered values to avoid multicollinearity before building into the regression models. In addition, moderation effects of gender and grade on PIU were tested by adding interaction terms to the model (ie, mother-/father-adolescent conflict × gender, mother-/father-adolescent conflict × grade, parental care/control × gender, parental care/control × grade). Furthermore, planned comparison analyses were used to test the difference between participants reporting no mother-/father-adolescent conflict and those reporting any verbal conflict, emotional abuse, and physical abuse from their mothers/fathers, with “0”=no score of conflict on all 3 questions regarding conflict; “1”=only score verbal conflict, no emotional or physical abuse; “2”=only score emotional abuse, no verbal conflict or physical abuse; “3”=only score physical abuse, no verbal conflict or emotional abuse; “4”=score all verbal conflicts, emotional abuse, and physical abuse.

### Ethics Approval

This project was approved by the Ethics Committee of the School of Public Health at Sun Yat-sen University, Guangzhou, China (number 2015-016). Written informed consent was provided by parents and assent was collected from all adolescent participants. This project also obtained administrative approval from the Queensland University of Technology Human Research Ethics Committee (Reference number: 108117).

## Results

### Preliminary Analyses

Table 1 shows the demographics of all the participants in this study. The mean age of participants (N=6552) was 13.51 (SD 2.93) years. In summary, 57.94% (3573/6166) of total participants were male; 43.71% (1734/3967) were from primary schools, with the remaining attending secondary school. Furthermore, 91.33% (5984/6552) were from nuclear families, and 10.82% (709/6552) of participants’ mothers and 15.55% (617/3697) of participant’s fathers had undergraduate degrees or above.

**Table 1:** Demographic characteristics of study participants (N=6552)^a^.

Demographic variables			Boys (n=3967), n (%)		Girls (n=2585), n (%)		Total sample, n (%)
**Grade**							
	Primary school	1734 (43.71)		1121 (43.37)		2855 (43.57)	
	Secondary school	2233 (56.29)		1464 (56.63)		3697 (56.43)	
**Family structure**							
	Nuclear families	3593 (90.57)		2391 (92.50)		5984 (91.33)	
	Others^a^	374 (9.43)		194 (7.50)		568 (8.67)	
**Maternal education level**							
	9th grade or less	2585 (65.16)		1637 (63.33)		4222 (64.44)	
	10-12th grade	950 (23.95)		671 (25.96)		1621 (24.74)	
	Undergraduate or above	432 (10.89)		277 (10.72)		709 (10.82)	
**Paternal education level**							
	9th grade or less	2164 (54.55)		1320 (51.06)		3484 (53.17)	
	10-12th grade	1186 (29.90)		835 (32.30)		2021 (30.85)	
	Undergraduate or above	617 (15.55)		430 (16.63)		1047 (15.98)	

^a^Includes but limited to, for example, single-parent families or separated families.

### Bivariate Correlation Analyses

Tables 2 and 3 show the means and SDs of the key variables categorized by sex. In addition, independent correlations between mother/father conflict, parental care/control, and PIU are presented in Table 3. The results show that mother-adolescent conflict and father-adolescent conflict were positively correlated with adolescent PIU. Moreover, parental care was negatively correlated with PIU, whereas parental control was positively correlated with PIU. These correlations were small in absolute magnitude (0.1<*r*<0.3).

**Table 2:** The mean and SD of variables.

Variables	Boys	Girls	Total
Mother-adolescent conflict	1.88 (1.79)	1.90 (1.93)	1.46 (1.96)
Father-adolescent conflict	1.56 (2.00)	1.29 (1.86)	1.91 (1.87)
Parental care	20.71 (5.26)	20.90 (5.29)	20.74 (5.28)
Parental control	12.61 (4.40)	12.11 (4.14)	12.48 (4.30)
Problematic internet use	73.32 (30.42)	64.32 (26.30)	69.47 (29.23)

**Table 3:** Correlation analysis (r).

Variables	Mother-adolescent conflict	Father-adolescent conflict	Parental care	Parental control	Problematic internet use
Mother-adolescent conflict	—^a^	0.49^b^	–0.42^b^	0.14^b^	0.26^b^
Father-adolescent conflict	—	—	–0.38^b^	0.17^b^	0.21^b^
Parental care	—	—	—	–0.21^b^	–0.25^b^
Parental control	—	—	—	—	0.12^b^
Problematic internet use	—	—	—	—	—

^a^Not applicable.

^b^*P*<.01.

### Multiple Linear Regression Analyses

Hypotheses 1 and 2 were tested using step 1 of a stepwise regression, with mother-adolescent conflict, father-adolescent conflict, parental care, and parental control as the independent variables and PIU as the dependent variable (Table 4). After the covariates were entered, higher mother-adolescent conflict, higher father-adolescent conflict, and higher parental control were independently associated with higher PIU (*β*=.145, *P*<.001; *β*=.077, *P*<.001; *β*=.055, *P*<.001, respectively), whereas higher parental care was independently associated with lower PIU (*β*=–.141, *P*<.001).

Hypothesis 3 was tested by adding interaction terms (see the “Statistical Analyses” section) to the aforesaid stepwise regression (also see Table 4). This analysis identified 2 moderating relationships only between parent-adolescent conflict and PIU. It shows that there was a significant association of adolescent-mother conflict and grade with the PIU reported by the adolescents (*β*=.045, *P*=.04), with the relationship being stronger in older students than in younger students. Another significant interaction effect was found between father-adolescent conflict and gender with the PIU reported (*β*=–.054, *P*=.02), with the relationship being stronger in male students than in female students.

Hypothesis 4 was tested using planned comparison analyses to compare adolescents reporting no mother-/father-adolescent conflict and those reporting any mother-/father-adolescent conflict (Table 5). For mother-adolescent conflict, the findings indicated that there was a significant difference between adolescents who reported no conflict and those reporting at least some level of verbal conflict, and emotional abuse (*P*<.001, respectively). However, no significant difference was found between those experiencing no conflict and those experiencing some level of physical abuse (*P*=.23). For father-adolescent conflict, there was a significant difference between those who reported no conflict and those reporting some verbal conflict (*P*<.001), emotional abuse (*P*<.001), and physical abuse (*P*=.02).

**Table 4:** Multiple linear regression analysis of the association of family-based relationships on adolescent’s levels of problematic internet use^a^.

Variables		Step 1			Step 2	
		*β*	t_10,6542_^b^	*β*		t_18,6534_^b^
Gender		–.139	–9.231^c^	–.143		–9.349^c^
Grade		.143	9.484^c^	.149		9.671^c^
Family structure		.036	2.075^d^	.036		2.069^e^
Maternal education level		.005	0.380	.005		–0.601
Paternal education level		.008	0.324	.008		0.377
Academic rank		.026	1.650	.028		0.307
**Mother-adolescent conflict (C)**		.145	8.134^c^	.136		4.586^c^
	C × gender	—^f^	—	–.029		–0.797
	C × grade	—	—	.045		2.018^d^
**Father-adolescent conflict (C)**		.077	4.376^c^	.102		3.402^e^
	C × gender	—	—	–.054		–2.314^d^
	C × grade	—	—	.012		0.534
**Parental care**		–.141	–8.315^c^	–.154		–5.964^c^
	Parental care × gender	—	—	–.003		0.094
	Parental care × grade	—	—	.022		0.979
**Parental control**		.055	3.590^c^	.060		2.360^d^
	Parental control × gender			.004		0.311
	Parental control × grade			–.013		–0.598

^a^*R*^2^=0.150 in step 2 and adjusted *R*^2^=0.145 (*P*<.05).

^b^2-tailed.

^c^*P*<.001.

^d^*P*<.05.

^e^*P*<.01.

^f^Not applicable.

**Table 5:** Descriptive statistics and planned comparison outcome of forms of mother-/father-adolescent conflict levels.

Group (R)		Mean (SD)	95% CI	Mean difference (R–C); SD
No conflict (reference)		59.61 (0.69)	58.24-60.98	
**Mother-adolescent conflict (C)**				
	Only verbal conflict	68.97 (0.72)	67.55-70.40	–9.36; 1.00^a^
	Only emotional abuse	66.85 (1.24)	64.40-69.30	–7.22; 1.43^a^
	Only physical abuse	65.03 (2.26)	60.62-69.48	–5.42; 2.36
	With all forms above	79.52 (0.89)	77.76-81.28	–19.91; 1.13^a^
No conflict (reference)		63.28 (0.52)	62.26-64.30	
**Father-adolescent conflict (C)**				
	Only verbal conflict	72.17 (1.03)	70.14-74.20	–8.89; 1.16^a^
	Only emotional abuse	70.45 (1.19)	68.11-72.70	–7.17; 1.30^a^
	Only physical abuse	68.67 (1.68)	65.39-71.95	–5.39; 1.76^b^
	With all forms above	79.95 (0.96)	78.07-81.80	–16.67; 1.09^a^

^a^*P*<.001.

^b^*P*<.05.

## Discussion

### Principal Findings

This study examined the associations between mother-/father-adolescent conflict (including verbal conflict, emotional abuse, and physical abuse), parenting styles (ie, parental care and parental control), and PIU in a large sample of Chinese adolescents after accounting for key demographics. Our findings were consistent with hypothesis 1, that is, greater mother-adolescent conflict and father-adolescent conflict were independently associated with higher PIU. The data were also consistent with hypothesis 2, that is, higher parental care was independently associated with lower PIU, whereas higher parental control was independently associated with higher PIU. The results were partially consistent with hypothesis 3, in that the association between father-adolescent conflict and PIU was stronger in male adolescents than in female adolescents, and the association of mother-adolescent conflict with PIU was stronger in older adolescents than in younger adolescents. The results for the last exploration found that, compared with adolescents reporting no conflict, adolescents experiencing more father-adolescent verbal conflict, emotional abuse, and physical abuse reported higher levels of PIU. When compared with those who reported no conflict with their mother, adolescents with more mother-adolescent verbal conflict and emotional abuse, but not physical abuse, reported higher levels of PIU.

The finding of an association between parenting styles of lower care/warmth or higher control and adolescent PIU is consistent with previous research studies [14-16], as are the findings of an association between adolescent-parent conflict and PIU [19,20]. Our findings add to the literature by identifying the unique effects of mother-/father-adolescent conflict and parental bonding (ie, parental care and parental control) on adolescent PIU after controlling for a range of demographics. This is important as the results highlight that there are multiple forms or qualities of the parent-adolescent relationship that need to be considered when identifying family risk factors for PIU, as well as when considering family interventions for PIU. A recent meta-analysis suggested that in addition to parental care and control, an authoritarian parental style was associated with adolescent PIU, whereas media-specific parenting styles and active mediation by parents were not associated with adolescent PIU [36]. As such, our findings add to the emerging body of evidence on the importance of identifying specific forms of parent-adolescent interactions that are toxic or protective for adolescent PIU.

Our study also found that gender moderated the association between father-adolescent conflict and PIU, with this association being stronger in male students than in female students. Given the gender stereotypes in traditional culture, boys are expected to be primary providers for their own family in adulthood and responsible for taking care of aged parents. Thus, there is a strong tendency for Chinese fathers to discipline their sons’ misbehaviors [37], which in turn may trigger more father-adolescent conflict among boys compared with girls. Similarly, a father’s overprotection/control may be more damaging to male adolescents than females [38]. In addition, grade moderated the association between mother-adolescent conflict and PIU, with this association being stronger in older students than in younger students. As adolescents grow older, they are exposed to the internet more, as well as subjected to higher academic expectations compared with younger adolescents [34]. In addition, older adolescents are more inclined to anxiety and depressed mood than younger adolescents [39]. This might be concerning for mothers as primary caregivers spending more time with adolescents [40,41]. Our findings point to the complexity in associations between the parent-adolescent relationship and PIU as moderated by the gender of the parent, as well as by the gender and age of the adolescent. These complexities are starting to be highlighted in other research. For example, there is meta-analytic evidence that the association between physical abuse and externalizing behaviors is stronger in female than in male Chinese children and adolescents, whereas the association between emotional abuse and externalizing behaviors is stronger in male and female Chinese children and adolescents [42]. In addition, there is meta-analytic evidence that a parenting style termed “restrictive mediation” is associated with PIU in older adolescents but not in younger adolescents [36]. Therefore, there is a need for further research to explore the impact of moderators such as the gender of the parent and adolescent, as well as the age of the adolescent on the associations between parent-adolescent relationships and PIU.

Interestingly, this study found that PIU was only related to paternal physical abuse and not maternal physical abuse. As paternal physical aggression is commonly associated with greater fear and intimidation than maternal physical aggression [43], it is possible that increases in adolescent anxiety add to the likelihood of adolescent problematic behaviors (including PIU) to escape, avoid, or seek support for family relationship distress [29,43-46]. In Chinese culture, fathers as disciplinary figures tend to harshly punish their children when behavioral expectations are not met and show less warmth toward children [47]. Although replication of these findings is needed, there is also a need for qualitative research to explore adolescents’ perceptions of why physical abuse from father but not mothers may be associated with adolescent PIU.

### Implications of Our Findings

Our findings have theoretical and practical implications for the prevention of and early intervention for adolescent PIU. In terms of theoretical implication, consistent with the Attachment Theory, this study demonstrated that the sense of connectedness or closeness adolescents have with their parents is important in understanding their maladaptive behaviors such as PIU. If this sense of connectedness or closeness is threatened by either a range of forms of interpersonal conflict or overcontrolling parenting style, then an adolescent is more likely to engage in PIU. Conversely, if this sense of connectedness or closeness to parents is enhanced by a parenting style exhibiting care and warmth, then an adolescent is less likely to engage in PIU. As such, our findings support a model of adolescent psychopathology based on Attachment Theory.

In terms of practical implications, the results support the expansion of current prevention strategies to include a focus on improving parenting styles (ie, increase parental care and decrease parental control) and managing parent-adolescent conflict and strengthening support for adolescents experiencing significant family conflict [18,48,49]. There is some preliminary evidence from a small study of 57 Chinese adolescents that a 14-session family-based group therapy can reduce PIU compared with an active control group [50]. This family-based therapy involved a range of therapeutic components (ie, promoting a supportive environment, studying how to correctly perceive and use the internet, changing cognition of themselves and establishing self-confidence, improving family functioning, and fostering hope for future recovery), and therefore, the extent to which the intervention addressed and improved the parent-adolescent relationship factors identified in this study remains unclear. A more direct application of the findings from our study to therapy would be the implementation of attachment-based family therapy for PIU. To date, no study has applied this therapy; however, there is strong evidence of its effectiveness for the treatment of adolescent depression, suicidal behavior, and anxiety [51]. Attachment-based family therapy is grounded in Attachment Theory and aims to reduce parent-adolescent conflict, repair interpersonal ruptures, and strengthen secure attachments between adolescents and parents [52]. As such, it has a strong theoretical alignment with the constructs identified in our study as risk and protective factors for PIU and we recommend that it be trialed in future research as an intervention for adolescent PIU.

### Strengths

The strengths of this study are the large sample size and a high response rate (6552/6638, 98.70%), which result in limited selection bias [53]. Another strength is that the proposed model controlled for several covariates that have previously been suggested to be strong predictors of adolescent PIU [54,55]. However, given the cross-sectional design, conclusions about causal relationships are not possible. Evidence suggests that adolescent’s behaviors (eg, having deviant peers) may also shape parenting style [13], and that parents may become frustrated, anxious/hostile, or rejecting in response to adolescent PIU, which then triggers parent-adolescent conflict and further escape/avoidance through internet use [56]. Further prospective research is therefore required to examine the possible bidirectional causality of the associations found in this study. In addition, as the focus of this study was on the relationship between adolescents and each parent individually, we combined nuclear families with single-parent families in the analyses. However, it will be useful for future studies to examine the associations identified in our study separately for nuclear families and single-parent families. It is also worth noting that the findings of this study were limited to general PIU rather than to specific PIU, for example, internet gaming addiction [57]. Moreover, the self-reported data are purely reflective of the adolescents’ perspective of their experiences with their parents and with PIU, leading to possible reporting biases. Previous research investigated whether there is consistency between mothers’ and children’s’ perceptions of parenting [58]. In addition, there is evidence that self-report measures of PIU may not be strongly congruent with both client log data [59] and clinical diagnostic interviews of adolescents [60]. Future research is therefore needed to confirm the findings from this study using multiple measures of adolescent-parent conflict, parental bonding, and PIU. We were unable to control for parental supervision and monitoring, and interparental conflict was not included as a covariate or as a possible risk factor. Future research should also consider including these variables in models of PIU given that they have been shown to be predictors of other adolescent risky behaviors [61-63]. Furthermore, it seems that maternal and paternal psychological and behavioral control have different effects on adolescent PIU [7]; therefore, future studies could subgroup parental care and control for a better understanding of how specific dimensions of parental bonding impact on adolescent PIU. Finally, given the relatively high comorbidity between adolescent PIU and externalizing behaviors such as conduct problems, hyperactivity, physical health problems, and depression [64-66], there is a need for future research building upon the findings of this study to compare the risk and protective factors for adolescent PIU and adolescent externalizing behaviors to identify unique and common risk/protective factors.

### Conclusions

In conclusion, higher levels of mother-adolescent conflict and father-adolescent conflict, higher levels of parental control, and lower levels of parental care were associated with higher PIU among Chinese adolescents. Furthermore, the effect for mother-adolescent conflict on PIU was stronger in older adolescents than in younger adolescents, whereas the effect for father-adolescent conflict on PIU was stronger in male adolescents than in female adolescents. These findings point to the potential utility of family-oriented education and early intervention for adolescent PIU by reducing verbal conflict as well as emotional and physical abuse, and strengthening parent-adolescent relationships through more affection from parents and less psychological control at home.

## Abbreviations

PBI: Parental Bonding Instrument
PIU: problematic internet use
WHO: World Health Organization
